# Association of Premenopausal Bilateral Oophorectomy With Restless Legs Syndrome

**DOI:** 10.1001/jamanetworkopen.2020.36058

**Published:** 2021-02-01

**Authors:** Nan Huo, Carin Y. Smith, Liliana Gazzuola Rocca, Walter A. Rocca, Michelle M. Mielke

**Affiliations:** 1 Division of Epidemiology, Department of Health Sciences Research, Mayo Clinic, Rochester, Minnesota; 2 Division of Biomedical Statistics and Informatics, Department of Health Sciences Research, Mayo Clinic, Rochester, Minnesota; 3 Department of Neurology, Mayo Clinic, Rochester, Minnesota; 4 Mayo Clinic Specialized Research Center of Excellence on Sex Differences, Mayo Clinic, Rochester, Minnesota

## Abstract

**Importance:**

Restless legs syndrome is a common neurologic disorder that is more prevalent in women than in men, and it has been suggested that female hormones may be involved in the disorder’s pathophysiology.

**Objective:**

To determine whether women who underwent premenopausal bilateral oophorectomy were at increased risk of restless legs syndrome.

**Design, Setting, and Participants:**

This cohort study was performed using data from the Mayo Clinic Cohort Study of Oophorectomy and Aging–2 for a population in Olmsted County, Minnesota. There were 1653 women who underwent premenopausal bilateral oophorectomy before the age of 50 years for a benign indication between 1988 and 2007 and 1653 age-matched women (of same age plus or minus 1 year) in a reference group. Follow-up was conducted until the end of the study period (ie, December 31, 2014). Data were analyzed from January to July 2020.

**Exposures:**

Undergoing bilateral oophorectomy, as shown in medical record documentation.

**Main Outcomes and Measures:**

Diagnosis of restless legs syndrome, as defined using *Diagnostic and Statistical Manual of Mental Disorders* (Fifth Edition) criteria, was recorded.

**Results:**

Among 3306 women, the median (interquartile range) age at baseline was 44.0 (40.0-47.0) years. Women who underwent bilateral oophorectomy, compared with women who did not undergo this procedure, had a greater number of chronic conditions at the index date (eg, 300 women [18.1%] vs 171 women [10.3%] with ≥3 chronic conditions; overall *P* < .001), were more likely to have obesity (576 women [34.8%] vs 442 women [27.1%]; overall *P* < .001), and were more likely to have a history of anemia of any type (573 women [34.7%] vs 225 women [13.6%]; *P* < .001), iron deficiency anemia (347 women [21.0%] vs 135 women [8.2%]; *P* < .001), and restless legs syndrome before the index date (32 women [1.9%] vs 14 women [0.8%]; *P* = .008). Women who underwent bilateral oophorectomy prior to natural menopause had a higher risk of restless legs syndrome after the index date compared with women in the reference group (120 diagnoses vs 74 diagnoses), with an adjusted hazard ratio (HR) of 1.44 (95% CI, 1.08-1.92; *P* = .01). After stratification by indication for the bilateral oophorectomy, there was an increased risk of restless legs syndrome among women without a benign ovarian condition (HR, 1.52; 95% CI, 1.03-2.25; *P* = .04) but not among women with a benign condition (HR, 1.25; 95% CI, 0.80-1.96; *P* = .34). Treatment with estrogen therapy through the age of 46 years in women who underwent bilateral oophorectomy at younger ages was not associated with a difference in risk.

**Conclusions and Relevance:**

This cohort study found that risk of restless legs syndrome was increased among women who underwent bilateral oophorectomy prior to menopause, especially those without a benign ovarian indication.

## Key Points

**Question:**

Is bilateral oophorectomy for a benign indication prior to natural menopause associated with risk of restless legs syndrome?

**Findings:**

In this cohort study of 3306 women, those who underwent bilateral oophorectomy prior to natural menopause had a higher risk of restless legs syndrome than those who did not undergo such surgery. The increased risk was independent of multiple chronic conditions present at baseline, was greater among women without a benign ovarian condition, and did not differ by estrogen therapy.

**Meaning:**

This study found that undergoing bilateral oophorectomy for a benign indication prior to natural menopause was associated with an increased risk of restless legs syndrome.

## Teaser

This cohort study assesses whether bilateral oophorectomy for a benign indication prior to natural menopause is associated with risk of restless legs syndrome.

## Introduction

Restless legs syndrome (RLS) is a neurological disorder associated with an irresistible desire to move the legs in response to uncomfortable feelings and unpleasant sensations.^1^ Although the mechanisms of RLS are not fully understood, some diseases and conditions, such as diabetes, kidney disease, Parkinson disease, iron deficiency, and pregnancy, are associated with RLS.^2,3^

Given the higher prevalence of RLS among women, the role of estrogen in the development of RLS has been examined. The prevalence of RLS is significantly increased among pregnant women compared with women who are not pregnant, and estrogen levels are higher during pregnancy; a woman’s risk of RLS later in life increases with each pregnancy.^3,4^ Thus, these studies suggest that high levels of estrogen may be associated with increased risk of RLS. By contrast, the prevalence of RLS is increased among women who have reached menopause, when the level of circulating estrogen decreases; however, the use of estrogen therapy in women who have reached menopause is not associated with reduced risk of RLS.^5^ Thus, additional studies are needed to examine risk factors associated with RLS among women.

Hysterectomy is one of the most common gynecologic surgical treatments in the United States.^6,7^ Women undergoing hysterectomy commonly are offered bilateral oophorectomy at the same time or later for ovarian cancer prevention.^6^ It is estimated that 1 in 8 US women have their ovaries removed before reaching natural menopause. There is increasing evidence that the ovarian hormone deprivation caused by bilateral oophorectomy is associated with accelerated aging and several neurological disorders.^8,9,10,11,12,13^ In this study, we used a population-based cohort design to investigate the association between premenopausal bilateral oophorectomy and risk of RLS.

## Methods

The Mayo Clinic and Olmsted Medical Center institutional review boards approved all research activities in this cohort study and waived informed consent as per Minnesota state privacy law, Statute §144.335.^14^ This study follows the Strengthening the Reporting of Observational Studies in Epidemiology (STROBE) reporting guideline.

### Data Source and Study Population

**Figure 1.**
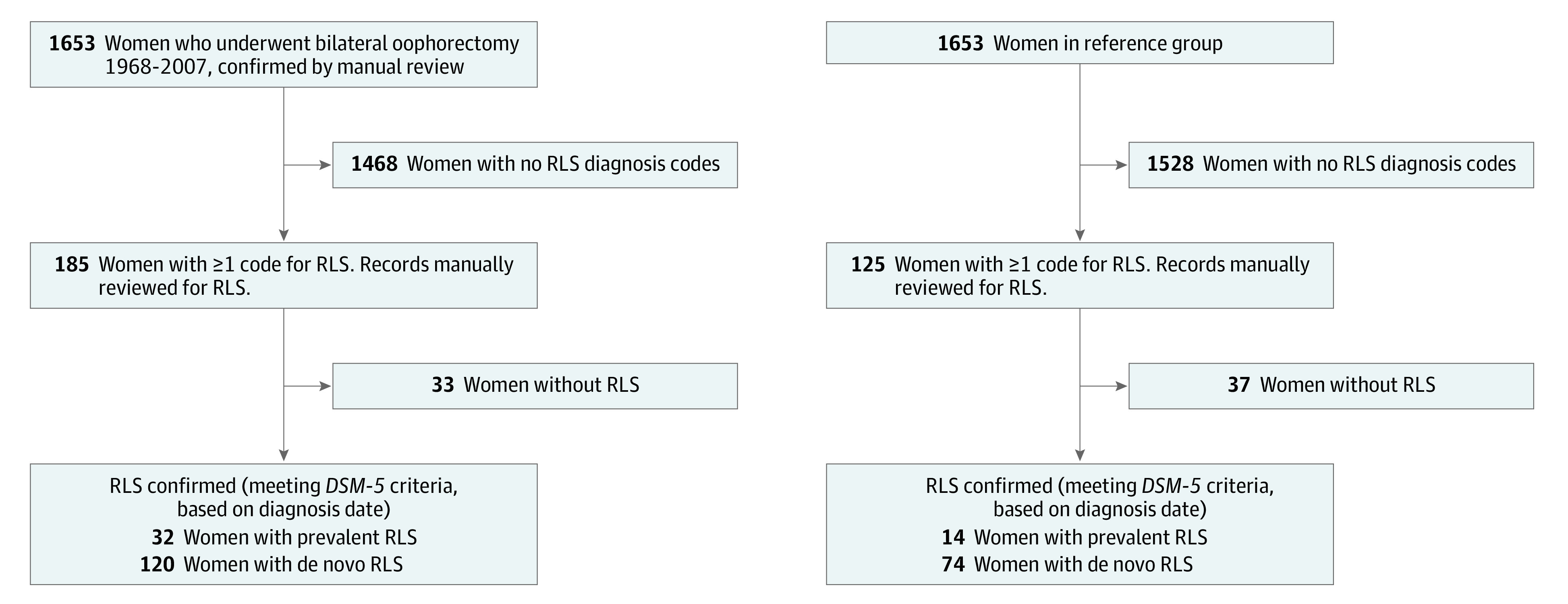
Flowchart of the 2 Study Groups The oophorectomy cohort was identified by reviewing the medical records for women with a procedure code for bilateral oophorectomy (including second unilateral oophorectomy). Women in the bilateral oophorectomy group were matched to a reference group of women without bilateral oophorectomy from the same population. For all women, a search was done for diagnostic codes for restless legs syndrome (RLS) at any time in life through the end of the study period (ie, December 31, 2014). After manual review of their complete medical records was conducted to confirm the diagnosis, women were classified as having RLS before or after the index date. *DSM-5* indicates *Diagnostic and Statistical Manual of Mental Disorders* (Fifth Edition).

The Mayo Clinic Cohort Study of Oophorectomy and Aging-2 (MOA-2) includes a cohort of 1653 women who underwent premenopausal bilateral oophorectomy from 1988 through 2007 and 1653 age-matched women in a reference group. The study sample and methods were previously described.^15,16,17^ Briefly, we included in MOA-2 all women who resided in Olmsted County, Minnesota, and received a procedure code from the *International Classification of Diseases, Ninth Revision *(*ICD-9*)^18^ for second unilateral (65.3 × and 65.4 ×) or bilateral (65.5 × and 65.6 ×) oophorectomy from January 1, 1988, to December 31, 2007, using the electronic indexes of the Rochester Epidemiology Project (REP) (Figure 1). We excluded women who had bilateral oophorectomy due to ovarian or other estrogen-sensitive cancers or due to a high genetic risk of ovarian cancer. The date of the surgical procedure was considered the index date. We used simple random sampling to match each woman who underwent bilateral oophorectomy 1:1 to a woman of the same age (plus or minus 1 year) as a reference at index date. Women in the reference group had not undergone bilateral oophorectomy before the index date. The cohorts were followed up passively using the REP medical record linkage system.^17,19,20,21,22^

### Main Outcome and Measures

The main outcome of interest was the cumulative incidence of RLS. The medical records for all women with *International Classification of Diseases, Eighth Revision *(*ICD-8*)^23^ or *ICD-9* diagnosis codes for RLS (333.94), myoclonus (333.2), and extrapyramidal disease (333.90 and 333.99) were reviewed by a physician (L.G.R.). The date the patient received a diagnosis of RLS meeting the criteria of the *Diagnostic and Statistical Manual of Mental Disorders* (Fifth Edition) (*DSM-5*)^24^ was recorded. To meet these criteria, a woman must report an urge to move her legs, usually accompanied by, or in response to, uncomfortable and unpleasant sensations in the legs, with all of the following characterizations true: (1) The urge to move the legs begins or worsens during periods of rest or inactivity. (2) The urge to move the legs is partially or totally relieved by movement. (3) The urge to move the legs is worse in the evening or at night than during the day or occurs only in the evening or at night. These symptoms must occur at least 3 times per week, must have persisted for at least 3 months, and must be accompanied by significant distress or impairment in social, occupational, educational, academic, behavioral, or other important areas of functioning. However, the symptoms should not be attributable to another mental disorder or medical condition (eg, arthritis), better explained by a behavioral condition (eg, positional discomfort), or attributable to physiological outcomes associated with a drug of abuse or a medication (eg, akathisia).^24^

### Other Variables

Demographic and clinical characteristics were determined at the index date and included age, education, race/ethnicity, household income, body mass index (BMI; calculated as weight in kilograms divided by height in meters squared), smoking status, and history of anemia of any type or iron deficiency anemia. Women needed to have at least 1 corresponding diagnostic code (from *ICD-8* or *ICD-9*) to be classified as having history of anemia of any type or history of iron deficiency anemia. In addition, we considered 18 of the 20 chronic conditions used by the US Department of Health and Human Services (HHS) to define multimorbidity: depression, anxiety, substance abuse disorders, dementia, schizophrenia or psychosis, hyperlipidemia, hypertension, diabetes, cardiac arrhythmias, coronary artery disease, stroke, congestive heart failure, arthritis, cancer, asthma, chronic obstructive pulmonary disease, osteoporosis, and chronic kidney disease. All chronic conditions were assessed using *ICD-8* or *ICD-9* diagnosis codes from the REP diagnostic indexes before the index date.^25^ Women needed to have at least 2 diagnostic codes in a given category separated by more than 30 days.^17^ Indications and pathology results for the bilateral oophorectomy were defined by the gynecologist and pathologist at the time of surgery. Extensive clinical information was manually abstracted from the medical records, including reproductive characteristics and use of systemic estrogen therapy after the date of the surgical procedure (ie, index date).

### Statistical Analysis

Descriptive characteristics at the index date were compared between the bilateral oophorectomy group and the reference group using χ^2^ tests. Women who met *DSM-5* criteria for RLS before the index date were excluded. Women were followed up from the index date to RLS diagnosis or were censored at the earliest of 3 end points: date of death, last visit with a REP provider, or the end of the study (ie, December 31, 2014). Data were analyzed from January to July 2020. Inverse probability weights derived from a logistic regression model were used to adjust for age at index date, calendar year, race/ethnicity (ie, White vs other race/ethnicity), BMI (ie, <30 vs ≥30), years of education (ie, ≤12, 13-16, or >16), quartiles of yearly household income (ie, <$42 000, $42 000-$56 999, $57 000-$71 999, or ≥$72 000), smoking status (ie, current or former smoker vs never smoked), and status at baseline for 18 chronic conditions. The balance of characteristics before and after inverse probability weighting is shown in the eFigure in the Supplement. After the inverse probability weight adjustment, the standardized differences for conditions or characteristics were below the recommended threshold of 0.10 (ie, negligible imbalance between the 2 cohorts). These adjustments were done overall and separately in each stratum to maximize the balance at the index date.

The cumulative risk of RLS after bilateral oophorectomy (or index) was calculated using the Kaplan-Meier method adjusted using inverse probability weighting. Differences between the 2 cohorts were measured using the absolute risk increase or absolute risk reduction obtained by subtracting the 2 absolute risks. Cox proportional hazards regression models using age as the time scale and inverse probability weighting were used to calculate the hazard ratios (HRs) and 95% CIs for RLS. The proportional hazards assumptions were checked using time-dependent covariates added to the Cox models and with graphical methods; assumptions were satisfied.^26^

The analyses were conducted in the overall sample and stratified by age at the date of the surgical procedure (ie, index date; ≤45 years vs 46-49 years), ovarian indication (ie, benign vs none), and estrogen therapy status (ie, age ≤45 years at index date and estrogen therapy continued up to age 46 years vs otherwise and age 46-49 years at index date and estrogen therapy continued up to age 50 years vs otherwise).

We performed 4 sets of sensitivity analyses. First, we censored at the date of surgery those women in the reference group who underwent bilateral oophorectomy after the index date and before age 50 years. Second, because some chronic diseases and medications are associated with RLS, we repeated the analyses after excluding women who at the index date, had any of the 18 HHS chronic diseases considered. Third, because iron deficiency anemia is associated with RLS,^2^ we repeated the analyses after excluding outcomes for women who had iron deficiency anemia at the time of RLS diagnosis. Fourth, we adjusted for history of anemia of any type at baseline.

Analyses were conducted using SAS statistical software version 9.4 (SAS Institute), and tests of statistical significance were conducted at the 2-tailed α-level of .05. Because of the risk of spurious significant findings due to multiple comparisons, all sensitivity analyses should be considered exploratory.

## Results

### Characteristics at Index Date

Among 3306 women, the median (interquartile range) age at baseline was 44.0 (40.0-47.0) years. Figure 1 shows detailed flowcharts for the 2 cohorts, and Table 1 displays characteristics for the oophorectomy and reference groups. There were 1653 women in the bilateral oophorectomy group and 1653 age-matched women in the reference group. The median (interquartile range [IQR]) length of follow-up was 14.5 (10.3-19.1) years among women who underwent bilateral oophorectomy and 14.4 (10.4-19.3) years among women in the reference group. Women who underwent bilateral oophorectomy, compared with women in the reference group, had a greater number of chronic conditions at the index date (eg, 300 women [18.1%] vs 171 women [10.3%] with ≥3 chronic conditions; overall *P* < .001), were more likely to have obesity (576 women [34.8%] vs 442 women [27.1%]; overall *P* < .001), and were more likely to have received a diagnosis of anemia of any type (573 women [34.7%] vs 225 women [13.6%]; *P* < .001), iron deficiency anemia (347 women [21.0%] vs 135 women [8.2%]; *P* < .001), and RLS (32 women [1.9%] vs 14 women [0.8%]; *P* = .008) before the date of the surgical procedure (ie, index date) (Table 1). Almost all of the women who underwent bilateral oophorectomy also underwent hysterectomy, either concurrent with oophorectomy (1472 women [89.1%]) or before oophorectomy (157 women [9.5%]).

**Table 1.** Baseline Characteristics

Characteristic	No. (%)		*P* value
	With bilateral oophorectomy (n = 1653)	Without bilateral oophorectomy (n = 1653)	
Age at index, y			
≤45	1031 (62.4)	1031 (62.4)	NA
46-49	622 (37.6)	622 (37.6)	
Age at index, median (IQR), y	44.0 (40.0-47.0)	44.0 (40.0-47.0)	NA
Race/ethnicity			
White	1611 (97.5)	1570 (95.0)	<.001
Black	18 (1.1)	29 (1.8)	
Asian	18 (1.1)	49 (3.0)	
Other	6 (0.4)	5 (0.3)	
Years of education			
≤12	526 (31.9)	478 (29.5)	.02
13-16	895 (54.2)	861 (53.2)	
>16	229 (13.9)	279 (17.2)	
Missing data^a^	3	35	
Income quartiles, $			
<42 000	414 (25.1)	406 (24.6)	.28
42 000-56 999	440 (26.7)	412 (25.0)	
57 000-71 999	419 (25.4)	412 (25.0)	
≥72 000	374 (22.7)	421 (25.5)	
Missing data^a^	6	2	
BMI category			
Underweight or in reference range (<25.0)	596 (36.1)	700 (42.9)	<.001
Overweight (25.0-29.9)	481 (29.1)	488 (29.9)	
Obesity (≥30.0)	576 (34.8)	442 (27.1)	
Missing data^a^	0	23	
Smoking status			
Never	897 (54.3)	957 (57.9)	.08
In past	393 (23.8)	377 (22.8)	
Current	363 (22.0)	319 (19.3)	
Ovarian indication^b^			
None	978 (59.2)	NA	NA
Benign	675 (40.8)	NA	
Chronic conditions at index, No.			
0	659 (39.9)	888 (53.7)	<.001
1	434 (26.3)	394 (23.8)	
2	260 (15.7)	200 (12.1)	
≥3	300 (18.1)	171 (10.3)	
Prevalent RLS meeting *DSM-5* criteria^c^	32 (1.9)	14 (0.8)	.008
History of anemia			
Anemia of any type^d^	573 (34.7)	225 (13.6)	<.001
Iron deficiency anemia^e^	347 (21.0)	135 (8.2)	<.001

Abbreviations: BMI, body mass index (calculated as weight in kilograms divided by height in meters squared); *DSM-5*, *Diagnostic and Statistical Manual of Mental Disorders* (Fifth Edition); IQR, interquartile range; NA, not applicable; RLS, restless legs syndrome.

^a^ In the regression models used to derive inverse probability weights, women with unknown education were assigned to the 12 years or fewer group, women with unknown income were assigned to the $42 000 to 56 999 quartile, and women with unknown BMI were assigned to the 30 or lower group.

^b^ The indication was listed by the gynecologist in the medical record at the time of oophorectomy. Benign ovarian conditions included benign tumors, cyst, or endometriosis in either ovary. No ovarian indication included women without a benign ovarian condition in either ovary. Historically, the terms *prophylactic*, *elective*, or *incidental* oophorectomy were used; however, we did not use these terms.

^c^ The odds ratio from a conditional logistic regression model was 2.29 (95% CI, 1.22-4.28; *P* = .01).

^d^ The odds ratio from a conditional logistic regression model was 3.68 (95% CI, 3.03-4.46; *P* < .001).

^e^ The odds ratio from a conditional logistic regression model was 3.04 (95% CI, 2.44-3.79; *P* < .001).

### Bilateral Oophorectomy and Risk of RLS

**Figure 2.**
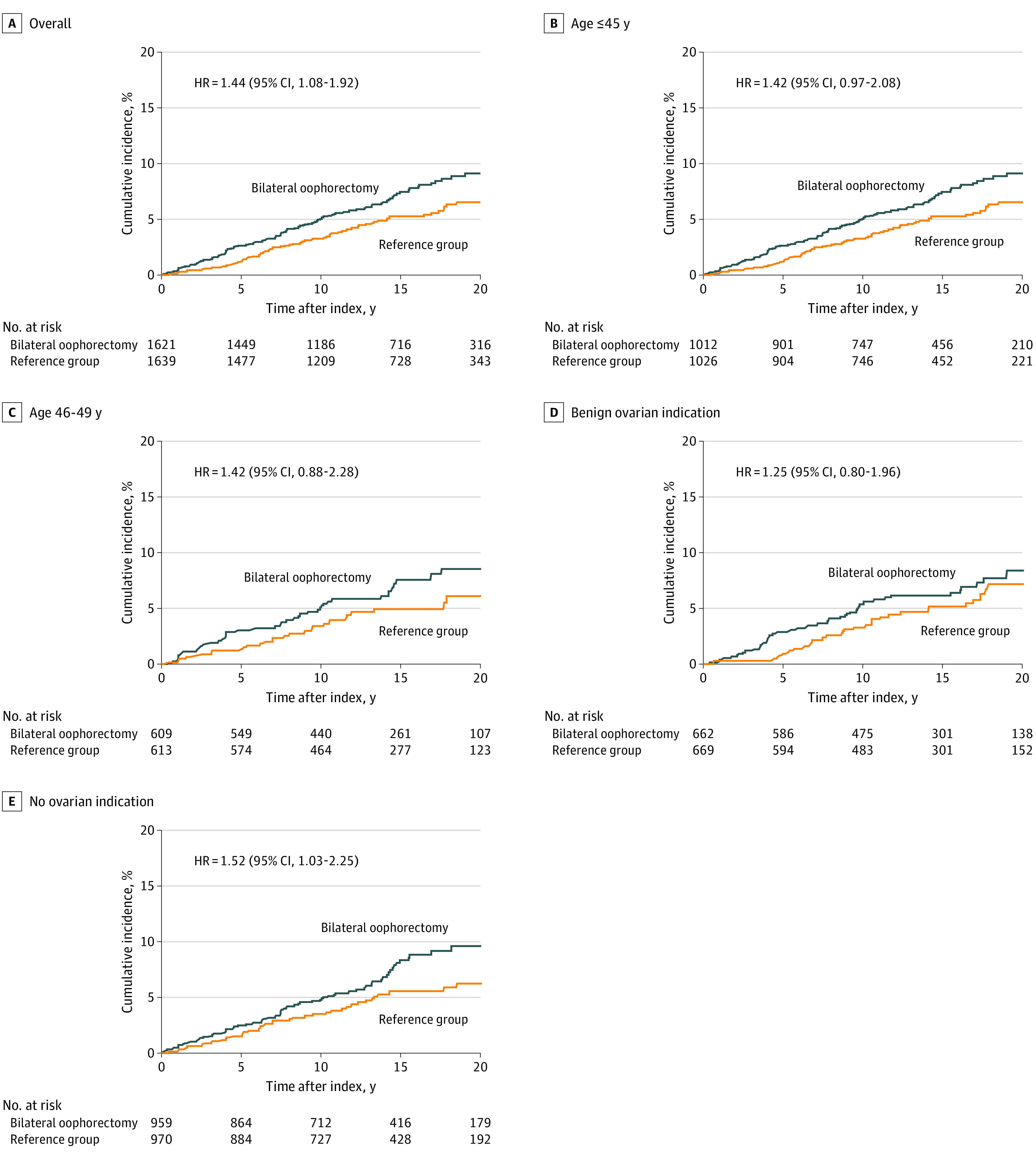
Risk of Restless Legs Syndrome (RLS) After Premenopausal Bilateral Oophorectomy Cumulative incidence curves for RLS in women who underwent bilateral oophorectomy compared with women who did not undergo this procedure (ie, the reference group), overall and in strata by age at index (ie, ≤45 years and 46-49 years) and surgical indication (ie, benign ovarian indication and no ovarian indication). HR indicates hazard ratio.

Of 1653 women in the bilateral oophorectomy group, 32 women (1.9%) were diagnosed with RLS that met *DSM-5* criteria before the index date and 120 women (9.1% cumulative incidence at 20 years) were diagnosed after the index date (Table 2; Figure 2). Of 1653 women in the reference group, 14 women (0.8%) were diagnosed with RLS before the index date and 74 women (6.8% cumulative incidence at 20 years) were diagnosed after the index date.

**Table 2.** Associations of Bilateral Oophorectomy With Incident RLS

Characteristic	Women with bilateral oophorectomy				Women without bilateral oophorectomy				Unweighted models^a^		Weighted models^b^	
	No. at risk	Person-years	Events, No.	Cumulative incidence at 20 y, % (95% CI)^c^	No. at risk	Person -years	Events, No.	Cumulative incidence at 20 y, % (95% CI)^c^	HR (95% CI)	*P* value	HR (95% CI)	*P* value
Overall	1621	22 666	120	9.1 (7.5-11.2)	1639	23 134	74	6.8 (5.3-8.7)	1.66 (1.25-2.21)	<.001	1.44 (1.08-1.92)	.01
Age, y												
≤45	1012	14 271	76	9.4 (7.3-12.1)	1026	14 335	46	6.9 (5.1-9.3)	1.67 (1.16-2.40)	.005	1.42 (0.97-2.08)	.07
46-49	609	8394	44	8.6 (6.1-11.9)	613	8799	28	7.0 (4.6-10.5)	1.65 (1.04-2.64)	.04	1.42 (0.88-2.28)	.15
≤45, with estrogen therapy^d^	636	7767	47	12.2 (8.4-17.5)	597	7308	32	7.4 (5.1-10.8)	1.38 (0.88-2.16)	.16	1.36 (0.86-2.14)	.18
≤45, without estrogen therapy	168	1573	6	7.9 (2.5-23.1)	160	1606	6	13.0 (6.1-26.7)	1.01 (0.33-3.09)	.99	0.60 (0.17-2.08)	.42
46-49, with estrogen therapy^e^	436	5677	31	11.2 (7.2-17.1)	418	5713	19	7.9 (4.9-12.7)	1.65 (0.93-2.91)	.09	1.42 (0.79-2.54)	.24
46-49, without estrogen therapy	154	1486	6	4.3 (1.6-11.7)	152	1561	6	3.8 (1.4-10.0)	1.05 (0.34-3.23)	.93	0.90 (0.28-2.85)	.86
Benign indication^f^	662	9299	47	8.4 (6.0-11.6)	669	9465	32	7.1 (4.9-10.3)	1.52 (0.99-2.34)	.06	1.25 (0.80-1.96)	.34
No ovarian indication^g^	959	13 366	73	9.6 (7.5-12.3)	970	13 669	42	6.8 (4.9-9.3)	1.78 (1.22-2.60)	.003	1.52 (1.03-2.25)	.04

Abbreviation: HR, hazard ratio.

^a^ HRs were calculated using Cox proportional hazards models with age as the time scale.

^b^ HRs were calculated using Cox proportional hazards models with age as the time scale and adjusted using inverse probability weights derived from a logistic regression model. Interactions by age, indication, and estrogen therapy were assessed using separate models. No significant interactions were found.

^c^ Cumulative risk of RLS at 20 years after bilateral oophorectomy (or index date) calculated using the Kaplan-Meier method. The estimates were adjusted using inverse probability weights derived from a logistic regression model.

^d^ Women who were receiving systemic estrogen therapy (only oral or transdermal) on their 46th birthday, after bilateral oophorectomy. Women who died or were lost to follow-up prior to their 46th birthday or had not reached age 46 years as of December 31, 2014, were not included in this analysis. Follow-up for these analyses was started when women reached age 46 years.

^e^ Women who were receiving systemic estrogen therapy (only oral or transdermal) on their 50th birthday, after bilateral oophorectomy. Women who died or were lost to follow-up prior to their 50th birthday or had not reached age 50 years as of December 31, 2014, were not included in this analysis. Follow-up for these analyses was started when women reached age 50 years.

^f^ The benign condition (eg, benign tumor, cysts, endometriosis) was listed by the gynecologist in the medical record at the time of bilateral oophorectomy but may not have been the sole indication for the surgery.

^g^ Women without an ovarian condition. Historically, the terms *prophylactic*, *elective*, or *incidental* bilateral oophorectomy were used; however, we did not use these terms.

Women who underwent bilateral oophorectomy had a higher risk of RLS in univariable models (unadjusted HR, 1.66; 95% CI, 1.25-2.21; *P* < .001) and in models adjusted using inverse probability weighting (HR, 1.44; 95% CI, 1.08-1.92; *P* = .01) (Table 2; Figure 2). The absolute risk of incident RLS 20 years after the index date was 9.1% (95% CI, 7.5%-11.2%) in the bilateral oophorectomy group and 6.8% (95% CI, 5.3%-8.7%) in the reference group (absolute risk increase, 2.3%). After stratification by indication, the HR was higher, but not significantly different, among women who underwent oophorectomy without an ovarian indication (HR, 1.52; 95% CI, 1.03-2.25; *P* = .04; absolute risk increase, 2.8%) compared with women with benign ovarian conditions (HR, 1.25; 95% CI, 0.80-1.96; *P* = .34; absolute risk increase, 1.3%; interaction *P* = .50). The most common ovarian indications were benign tumor, cyst, or endometriosis.

Among women who underwent bilateral oophorectomy before age 46 years, the HR of RLS was not significantly different for women who did not take estrogen therapy or stopped before their 46th birthday compared with women who received estrogen therapy through their 46th birthday. Similarly, among women who underwent bilateral oophorectomy between the ages of 46 and 49 years, the HR of RLS was not significantly different among women who did not take estrogen or stopped before their 50th birthday compared with women who received estrogen through their 50th birthday.

### Sensitivity Analyses

First, we censored women in the reference group who underwent bilateral oophorectomy after the index date, but the results did not change (eTable 1 in the Supplement). Second, we excluded women who, at the index date, had any of the 18 HHS-defined chronic diseases we considered, but the results were not changed (eTable 2 in the Supplement). Third, analyses excluding outcomes for women who had iron deficiency anemia at the time of RLS diagnosis showed similar results as the analysis with those outcomes included (eTable 3 in the Supplement). Fourth, when we adjusted for history of anemia of any type at baseline, the association of bilateral oophorectomy with risk of RLS diagnosis remained (eTable 4 in the Supplement).

We also examined the association of anemia of any type and iron deficiency anemia with bilateral oophorectomy before and after the index date (ie, date of surgery). Before the index date, anemia of any type (odds ratio [OR], 3.68; 95% CI, 3.03-4.46; *P* < .001) and iron deficiency anemia (OR, 3.04; 95% CI, 2.44-3.79; *P* < .001) were associated with bilateral oophorectomy using conditional logistic regression models. However, after the index date, there was no association between bilateral oophorectomy and risk of anemia of any type (HR, 1.13; 95% CI, 0.97-1.31; *P* = .11) or risk of iron deficiency anemia (HR, 1.06; 95% CI, 0.87-1.30; *P* = .55).

## Discussion

In this cohort study, we investigated the association between premenopausal bilateral oophorectomy and risk of RLS using a large population-based group of 3306 women followed up longitudinally through a medical record linkage system. Our results suggest that the incidence of RLS was higher in women who underwent premenopausal bilateral oophorectomy compared with age-matched women who did not undergo this procedure. Moreover, the increase in risk was greatest among women who had no ovarian indication for surgery. Estrogen therapy was not associated with a significant change in the risk association. This study has several strengths. The bilateral oophorectomy and reference cohorts were representative of a well-defined population with up to 27 years of follow-up. Furthermore, bilateral oophorectomy and RLS were identified through medical record abstraction, thus limiting recall bias.

Studies from 2007,^13^ 2012,^12^ and 2014^27^ found that prophylactic premenopausal bilateral oophorectomy was associated with an increased risk of dementia, and a 2008 study^10^ found an association with Parkinson disease. Our study extends this research to also show an association between bilateral oophorectomy and risk of RLS, another common neurodegenerative condition. The prevalence of RLS in the general population ranges from 2% to 10%.^28,29,30,31^ The higher cumulative incidence we found of RLS among women with premenopausal bilateral oophorectomy compared with women in the reference group is worthy of attention from clinicians. A 2002^32^ and a 2012^33^ study found an increase in the prevalence of RLS associated with increasing age. One possible explanation for the increased risk of RLS among women who underwent premenopausal bilateral oophorectomy before 50 years of age is that the abrupt estrogen deprivation caused by the removal of both ovaries is associated with accelerated aging. Indeed, women who have undergone premenopausal bilateral oophorectomy have an increased risk of accelerated aging^16^ and dementia.^10,11,13^

The association of premenopausal bilateral oophorectomy with changes in the dopaminergic system is another possible mechanism associated with the increased risk of RLS. Some studies have suggested that RLS is associated with basal ganglia dopamine levels, which contribute to the control of muscle activity and movement; treatments currently prescribed for RLS include dopamine agonists and alpha-2-delta calcium channel ligands.^34,35^ Notably, estrogen is essential for maintaining nigrostriatal dopaminergic neurons.^36,37^ Women who underwent premenopausal bilateral oophorectomy, especially before age 46 years, have a higher risk of neurodegenerative diseases.^10^ In addition to the direct association of estrogen with changes in the dopaminergic system, estrogen may be indirectly associated through changes in vitamin D metabolism.^38^ For example, the abrupt decline in estrogen levels after premenopausal bilateral oophorectomy could be associated with vitamin D deficiency.^39^

Women in our study who underwent premenopausal bilateral oophorectomy had a greater number of chronic conditions at the index date compared with women in the reference group, and some of these conditions have been associated with increased risk of RLS.^40^ In addition, patients with multiple chronic conditions usually need several different medications, which may themselves be associated with RLS symptoms. For example, tricyclic antidepressants and selective serotonin reuptake inhibitors are associated with an increased risk of RLS,^41^ likely associated with increased serotonin and norepinephrine activity and reduced dopaminergic activity.^42^ Because chronic health conditions are associated with bilateral oophorectomy and RLS, we conducted a sensitivity analysis excluding women who, at the index date, had any of 18 chronic conditions we considered, but the results were unchanged.

We did not observe a significant association between estrogen use through an individual’s 46th birthday and risk of RLS among women who underwent oophorectomy before age 46 years. This finding is in contrast to previous studies that suggested that estrogen therapy use was associated with an increased risk of developing RLS.^43,44^ However, there are complex associations among estrogens, dopamine, and movement disorders, and much remains unknown.^45,46^ For example, a 2007 study^47^ suggested that high estrogen levels are associated with a reduced risk of RLS. Therefore, studies examining the balance of risks and benefits of estrogen use after bilateral oophorectomy against the risk of subsequent RLS are needed.

### Limitations

This study has several limitations. First, participants were predominantly White, and the study included only women residing in Olmsted County, Minnesota. Thus, results may not be generalizable to other populations. Second, we did not have information on blood iron levels prior to the date of surgery or during follow-up. However, we studied the effect of iron indirectly, using anemia of any type and iron deficiency anemia as surrogate variables. Although both were associated with bilateral oophorectomy at the index date (ie, date of surgery), bilateral oophorectomy was not associated with increased risk of de novo anemia of any type or of iron deficiency anemia during follow-up. Among women who developed RLS, the frequency of concomitant iron deficiency anemia (ie, iron deficiency anemia present at the time of RLS diagnosis) was lower in the oophorectomy group compared with the reference group. This difference was due to the removal of the uterus in conjunction with the bilateral oophorectomy. A reduction of uterine bleeding will result in a reduced risk of RLS associated with iron deficiency anemia. These results suggest that iron deficiency anemia cannot explain the association between bilateral oophorectomy and increased risk of subsequent RLS.

Third, the retrospective, observational nature of the study design limits causal inference. Women with clinically recognized multiple chronic conditions may be more likely to undergo bilateral oophorectomy in the context of a hysterectomy. However, we performed a sensitivity analysis to reduce such bias by excluding women with a documented history of chronic conditions before the index date, and the results did not change. Fourth, the sample size and the corresponding statistical power were inadequate to consider some specific strata of estrogen therapy separately.

## Conclusions

This large cohort study found that the incidence of RLS was higher among women who underwent premenopausal bilateral oophorectomy compared with women who did not undergo this procedure. Physicians treating women with premenopausal bilateral oophorectomy need to be aware of their patients’ risk of RLS and create treatment-monitoring plans. These findings agree with the results of other studies showing associations of premenopausal bilateral oophorectomy with the risk of multiple chronic conditions. In conjunction, those results may help women at average risk of ovarian cancer to better evaluate the risk-benefit ratio of undergoing the bilateral oophorectomy prior to natural menopause for ovarian cancer prevention.

## Notes

Supplement. **eTable 1.** Association of Bilateral Oophorectomy With Incident Restless Legs Syndrome Meeting *Diagnostic and Statistical Manual of Mental Disorders* (Fifth Edition) Criteria **eTable 2.** Association of Bilateral Oophorectomy With Incident Restless Legs Syndrome Meeting *Diagnostic and Statistical Manual of Mental Disorders* (Fifth Edition) Criteria in Women Without Any of 18 Chronic Conditions at Baseline **eTable 3.** Association of Bilateral Oophorectomy With Incident Restless Legs Syndrome (RLS) Meeting *Diagnostic and Statistical Manual of Mental Disorders* (Fifth Edition) Criteria Excluding RLS With Concurrent Iron Deficiency Anemia at Diagnosis Date **eTable 4.** Association of Bilateral Oophorectomy With Incident Restless Legs Syndrome Meeting *Diagnostic and Statistical Manual of Mental Disorders* (Fifth Edition) Criteria Including Adjustment for History of Anemia of Any Type at Baseline **eFigure.** Balance of Characteristics at Baseline Obtained Using Inverse Probability Weights
