# Supplementary material for: Association of Premenopausal Bilateral Oophorectomy With Restless Legs Syndrome
Source: JAMA Netw Open. 2021 Feb 1;4(2):e2036058. doi: 10.1001/jamanetworkopen.2020.36058 (PMC7851733; doi:10.1001/jamanetworkopen.2020.36058)
Supplement: Supplement. — eTable 1. Association of Bilateral Oophorectomy With Incident Restless Legs Syndrome Meeting Diagnostic and Statistical Manual of Mental Disorders (Fifth Edition) Criteria eTable 2. Association of Bilateral Oophorectomy With Incident Restless Legs Syndrome Meeting Diagnostic and Statistical Manual of Mental Disorders (Fifth Edition) Criteria in Women Without Any of 18 Chronic Conditions at Baseline eTable 3. Association of Bilateral Oophorectomy With Incident Restless Legs Syndrome (RLS) Meeting Diagnostic and Statistical Manual of Mental Disorders (Fifth Edition) Criteria Excluding RLS With Concurrent Iron Deficiency Anemia at Diagnosis Date eTable 4. Association of Bilateral Oophorectomy With Incident Restless Legs Syndrome Meeting Diagnostic and Statistical Manual of Mental Disorders (Fifth Edition) Criteria Including Adjustment for History of Anemia of Any Type at Baseline eFigure. Balance of Characteristics at Baseline Obtained Using Inverse Probability Weights [file jamanetwopen-e2036058-s001.pdf]

Supplemental Online Content

Huo N, Smith CY, Gazzuola Rocca L, Rocca WA, Mielke MM. Association of premenopausal bilateral oophorectomy with restless legs syndrome. *JAMA Netw Open*. 2021;4(2):e2036058. doi:10.1001/jamanetworkopen.2020.36058

eTable 1. Association of Bilateral Oophorectomy With Incident Restless Legs Syndrome Meeting *Diagnostic and Statistical Manual of Mental Disorders* (Fifth Edition) Criteria

eTable 2. Association of Bilateral Oophorectomy With Incident Restless Legs Syndrome Meeting *Diagnostic and Statistical Manual of Mental Disorders* (Fifth Edition) Criteria in Women Without Any of 18 Chronic Conditions at Baseline

eTable 3. Association of Bilateral Oophorectomy With Incident Restless Legs Syndrome (RLS) Meeting *Diagnostic and Statistical Manual of Mental Disorders* (Fifth Edition) Criteria Excluding RLS With Concurrent Iron Deficiency Anemia at Diagnosis Date

eTable 4. Association of Bilateral Oophorectomy With Incident Restless Legs Syndrome Meeting *Diagnostic and Statistical Manual of Mental Disorders* (Fifth Edition) Criteria Including Adjustment for History of Anemia of Any Type at Baseline

eFigure. Balance of Characteristics at Baseline Obtained Using Inverse Probability Weights

This supplemental material has been provided by the authors to give readers additional information about their work.

© 2021 Huo N et al. *JAMA Network Open.*

eTable 1. Association of Bilateral Oophorectomy With Incident Restless Legs Syndrome Meeting *Diagnostic and Statistical Manual of Mental Disorders* (Fifth Edition) Criteria

	**Women with bilateral oophorectomy**				**Women without bilateral oophorectomy[a]**				**Unweighted models[b]**		**Weighted models[c]**	
**Strata**	**No. at risk**	**Person-years**	**No. of events**	**Cumulative incidence at 20 y,[d] % (95% CI)**	**No. at risk**	**Person-years**	**No. of events**	**Cumulative incidence at 20 y,[d] % (95% CI)**	**HR (95% CI)**	***P* value**	**HR (95% CI)**	***P* value**
Overall	1621	22 666	120	9.1 (7.5-11.2)	1639	22 113	70	6.9 (5.4-8.9)	**1.68 (1.25-2.25)**	**<.001**	**1.45 (1.07-1.96)**	**.02**
Age ≤45 y	1012	14 271	76	9.4 (7.3-12.1)	1026	13 496	43	7.1 (5.2-9.7)	**1.67 (1.15-2.43)**	**.007**	1.42 (0.96-2.09)	.08
Age 46-49 y	609	8394	44	8.6 (6.1-11.9)	613	8617	27	6.9 (4.5-10.6)	**1.68 (1.04-2.71)**	**.03**	1.44 (0.88-2.34)	.14
Benign indication[e]	662	9299	47	8.4 (6.0-11.6)	669	8850	29	7.3 (5.0-10.7)	1.56 (0.99-2.48)	.06	1.27 (0.79-2.05)	.32
No ovarian indication[f]	959	13 366	73	9.6 (7.5-12.3)	970	13 263	41	6.9 (5.0-9.5)	**1.77 (1.20-2.59)**	**.004**	**1.51 (1.01-2.24)**	**.04**
Age ≤45 y, estrogen therapy[g]	636	7767	47	12.2 (8.4-17.5)	580	6700	29	7.6 (5.1-11.2)	1.40 (0.88-2.22)	.15	1.36 (0.85-2.17)	.20
Age ≤45 y, No estrogen therapy	168	1573	6	7.9 (2.5-23.1)	157	1523	6	13.9 (6.4-28.4)	0.96 (0.31-2.94)	.94	0.56 (0.16-1.97)	.37
Age 46-49 y, estrogen therapy[h]ET[g]	436	5677	31	11.2 (7.2-17.1)	408	5577	19	8.1 (5.0-13.0)	1.61 (0.91-2.85)	.10	1.38 (0.77-2.47)	.28
Age 46-49 y, No estrogen therapy	154	1486	6	4.3 (1.6-11.7)	149	1530	5	2.4 (0.8-7.1)	1.22 (0.38-3.99)	.74	1.10 (0.33-3.72)	.87

Abbreviations: CI, confidence interval; HR, hazard ratio.

[a]Women in reference group were censored at the time of bilateral oophorectomy after index.

[b] HRs were calculated using Cox proportional hazards models with age as the time scale.

[c] HRs were calculated using Cox proportional hazards models with age as the time scale and adjusted using inverse probability weights derived from a logistic regression model. Interactions by age, indication, and estrogen therapy were assessed using separate models. No significant interactions were found,

© 2021 Huo N et al. *JAMA Network Open.*

[d] Cumulative risk of restless legs syndrome at 20 years after bilateral oophorectomy (or index) calculated using the Kaplan-Meier method. The estimates were adjusted using inverse probability weights derived from a logistic regression model.

[e] The benign condition (eg, benign tumor, cysts, endometriosis) was listed by the gynecologist in the medical record at the time of bilateral oophorectomy, but may not have been the sole indication for the surgery.

[f] Women without a benign ovarian condition. Historically, the terms *prophylactic*, *elective*, or *incidental* bilateral oophorectomy were used; however, we did not use these terms.

[g] Women who were receiving systemic estrogen therapy (only oral or transdermal) on their 46th birthday, after bilateral oophorectomy. Women who died or were lost to follow-up prior to their 46th birthday, or had not reached age 46 years as of December 31, 2014 were not included in this analysis. Follow-up for these analyses was started when women reached age 46 years.

[h] Women who were receiving systemic estrogen therapy (only oral or transdermal) on their 50th birthday, after bilateral oophorectomy. Women who died or were lost to follow-up prior to their 50th birthday, or had not reached age 50 years as of December 31, 2014 were not included in this analysis. Follow-up for these analyses was started when women reached age 50 years.

© 2021 Huo N et al. *JAMA Network Open.*

eTable 2. Association of Bilateral Oophorectomy With Incident Restless Legs Syndrome Meeting *Diagnostic and Statistical Manual of Mental Disorders* (Fifth Edition) Criteria in Women Without Any of 18 Chronic Conditions at Baseline[a]

	**Women with bilateral oophorectomy**				**Women without bilateral oophorectomy**				**Unweighted models[b]**		**Weighted models[c]**	
**Strata**	**No. at risk**	**Person-years**	**No. of events**	**Cumulative incidence at 20 y,[d] % (95% CI)**	**No. at risk**	**Person-years**	**No. of events**	**Cumulative incidence at 20 y,[d] % (95% CI)**	**HR (95% CI)**	***P* value**	**HR (95% CI)**	***P* value**
Overall	655	9884	37	7.3 (5.1-10.3)	879	12 782	24	4.1 (2.6-6.3)	**1.95 (1.17-3.25)**	**.01**	**1.92 (1.14-3.21)**	**.01**
Age ≤45 y	417	6379	22	7.3 (4.6-11.3)	588	8426	17	3.8 (2.2-6.6)	1.68 (0.89-3.14)	.11	1.72 (0.91-3.24)	.10
Age 46-49 y	238	3505	15	6.7 (3.6-12.1)	291	4356	7	4.8 (2.2-10.0)	**2.61 (1.06-6.39)**	**.04**	2.11 (0.85-5.24)	.11
Benign indication[e]	288	4391	12	5.4 (2.9-10.0)	382	5585	13	5.0 (2.7-9.2)	1.15 (0.52-2.54)	.72	1.11 (0.51-2.43)	.79
No ovarian indication[f]	367	5493	25	8.7 (5.7-13.3)	497	7197	11	3.1 (1.6-5.9)	**2.91 (1.45-5.84)**	**.003**	**3.04 (1.49-6.20)**	**.002**

Abbreviations: CI, confidence interval; DSM-V, Diagnostic and Statistical Manual of Mental Disorders, 5th edition; HR, hazard ratio.

[a] Analyses stratified by estrogen therapy are not reported because of small numbers.

[b] HRs were calculated using Cox proportional hazards models with age as the time scale.

[c] HRs were calculated using Cox proportional hazards models with age as the time scale and adjusted using inverse probability weights derived from a logistic regression model. Interactions by age and indication were assessed using separate models. No significant interactions were found.

[d] Cumulative risk of restless legs syndrome at 20 years after bilateral oophorectomy (or index) calculated using the Kaplan-Meier method. The estimates were adjusted using inverse probability weights derived from a logistic regression model.

[e] The benign condition (eg, benign tumor, cysts, endometriosis) was listed by the gynecologist in the medical record at the time of bilateral oophorectomy, but may not have been the sole indication for the surgery.

[f] Women without a benign ovarian condition. Historically, the terms *prophylactic*, *elective*, or *incidental* bilateral oophorectomy were used; however, we did not use these terms.

© 2021 Huo N et al. *JAMA Network Open.*

eTable 3. Association of Bilateral Oophorectomy With Incident Restless Legs Syndrome (RLS) Meeting *Diagnostic and Statistical Manual of Mental Disorders* (Fifth Edition) Criteria Excluding RLS With Concurrent Iron Deficiency Anemia at Diagnosis Date[a]

	**Women with bilateral oophorectomy**				**Women without bilateral oophorectomy**				**Unweighted models[b]**		**Weighted models[c]**	
**Strata**	**No. at risk**	**Person-years**	**No. of events**	**Cumulative incidence at 20 y,[d] % (95% CI)**	**No. at risk**	**Person-years**	**No. of events**	**Cumulative incidence at 20 y,[d] % (95% CI)**	**HR (95% CI)**	***P* value**	**HR (95% CI)**	***P* value**
Overall	1638	23 241	73	5.5 (4.2-7.1)	1643	23 408	39	3.9 (2.8-5.4)	**1.90 (1.30-2.78)**	**<.001**	**1.58 (1.07-2.35)**	**.02**
Age ≤45 y	1022	14 629	44	5.5 (3.9-7.6)	1027	14 509	22	3.8 (2.5-5.8)	**2.00 (1.20-3.34)**	**.008**	1.60 (0.94-2.74)	.08
Age 46-49 y	616	8612	29	5.5 (3.6-8.2)	616	8898	17	4.1 (2.5-6.9)	1.77 (0.99-3.17)	.06	1.51 (0.83-2.74)	.18
Benign indication[e]	666	9490	28	4.9 (3.2-7.7)	671	9621	13	3.8 (2.2-6.6)	**2.25 (1.20-4.23)**	**.01**	1.62 (0.83-3.16)	.16
No ovarian indication[f]	972	13 750	45	5.8 (4.2-8.0)	972	13 786	26	4.1 (2.8-6.0)	**1.74 (1.07-2.82)**	**.02**	1.46 (0.88-2.41)	.14
Age ≤45 y, estrogen therapy[g]	643	7978	31	8.2 (5.2-12.9)	599	7440	14	3.2 (1.8-5.7)	**2.06 (1.10-3.87)**	**.02**	**2.06 (1.09-3.90)**	**.03**
Age ≤45 y, No estrogen therapy	173	1633	1	1.2 (0.2-6.1)	161	1623	4	10.5 (4.3-24.4)	0.25 (0.03-2.20)	.21	0.24 (0.03-2.31)	.22
Age 46-49 y, estrogen therapy[h]	440	5820	20	7.8 (4.4-13.7)	421	5779	12	5.1 (2.8-9.2)	1.66 (0.81-3.40)	.16	1.40 (0.67-2.92)	.37
Age 46-49 y, No estrogen therapy	158	1543	4	3.0 (0.8-10.7)	153	1590	3	1.9 (0.4-8.8)	1.40 (0.32-6.08)	.65	1.12 (0.25-5.03)	.88

Abbreviations: CI, confidence interval; HR, hazard ratio.

[a] Women with RLS who had iron deficiency anemia at the time of RLS diagnosis were not included as outcomes. These women were censored at follow-up, similar to women who did not develop RLS.

[b] Hazard ratios were calculated using Cox proportional hazards models with age as the time scale.

© 2021 Huo N et al. *JAMA Network Open.*

[c] Hazard ratios were calculated using Cox proportional hazards models with age as the time scale and adjusted using inverse probability weights derived from a logistic regression model. Interactions by age, indication, and estrogen therapy were assessed using separate models. No significant interactions were found.

[d] Cumulative risk of RLS at 20 years after bilateral oophorectomy (or index) calculated using the Kaplan-Meier method. The estimates were adjusted using inverse probability weights derived from a logistic regression model.

[e] The benign condition (eg, benign tumor cysts, endometriosis) was listed by the gynecologist in the medical record at the time of bilateral oophorectomy, but may not have been the sole indication for the surgery.

[f] Women without a benign ovarian condition. Historically, the terms *prophylactic*, *elective*, or *incidental* bilateral oophorectomy were used; however, we did not use these terms.

[g] Women who were receiving systemic estrogen therapy (only oral or transdermal) on their 46th birthday, after bilateral oophorectomy. Women who died or were lost to follow-up prior to their 46th birthday, or had not reached age 46 years as of December 31, 2014 were not included in this analysis. Follow-up for these analyses was started when women reached age 46 years.

[h] Women who were receiving systemic estrogen therapy (only oral or transdermal) on their 50th birthday, after bilateral oophorectomy. Women who died or were lost to follow-up prior to their 50th birthday, or had not reached age 50 years as of December 31, 2014 were not included in this analysis. Follow-up for these analyses was started when women reached age 50 years.

© 2021 Huo N et al. *JAMA Network Open.*

eTable 4. Association of Bilateral Oophorectomy With Incident Restless Legs Syndrome Meeting *Diagnostic and Statistical Manual of Mental Disorders* (Fifth Edition) Criteria Including Adjustment for History of Anemia of Any Type at Baseline

	**Women with bilateral oophorectomy**				**Women without bilateral oophorectomy**				**Unweighted models**[a]		**Weighted models**[b]	
**Strata**	**No. at risk**	**Person-years**	**No. of events**	**Cumulative incidence at 20 y,[c] % (95% CI)**	**No. at risk**	**Person-years**	**No. of events**	**Cumulative incidence at 20 y,[c] % (95% CI)**	**HR (95% CI)**	***P* value**	**HR (95% CI)**	***P* value**
Overall	1621	22 666	120	9.1 (7.5-11.2)	1639	23 134	74	6.8 (5.3-8.7)	**1.60 (1.19-2.14)**	**.002**	**1.39 (1.03-1.88)**	**.03**
Age ≤45 y	1012	14 271	76	9.4 (7.3-12.1)	1026	14 335	46	6.9 (5.1-9.3)	**1.64 (1.14-2.37)**	**.008**	1.41 (0.96-2.08)	.08
Age 46-49 y	609	8394	44	8.6 (6.1-11.9)	613	8799	28	7.0 (4.6-10.5)	1.51 (0.91-2.49)	.11	1.32 (0.79-2.20)	.28
Benign indication[d]	662	9299	47	8.4 (6.0-11.6)	669	9465	32	7.1 (4.9-10.3)	1.50 (0.97-2.32)	.07	1.23 (0.78-1.94)	.37
No ovarian indication[e]	959	13 366	73	9.6 (7.5-12.3)	970	13 669	42	6.8 (4.9-9.3)	**1.68 (1.13-2.50)**	**.01**	1.44 (0.95-2.19)	.09
Age ≤45 y, estrogen therapy[f]	636	7767	47	12.2 (8.4-17.5)	597	7308	32	7.4 (5.1-10.8)	1.41 (0.90-2.22)	.13	1.41 (0.89-2.22)	.14
Age ≤45 y, No estrogen therapy	168	1573	6	7.9 (2.5-23.1)	160	1606	6	13.0 (6.1-26.7)	0.62 (0.24-1.58)	.31	0.37 (0.13-1.08)	.07
Age 46-49 y, estrogen therapy[g]	436	5677	31	11.2 (7.2-17.1)	418	5713	19	7.9 (4.9-12.7)	1.55 (0.83-2.89)	.17	1.35 (0.71-2.57)	.36
Age 46-49 y, No estrogen therapy	154	1486	6	4.3 (1.6-11.7)	152	1561	6	3.8 (1.4-10.0)	1.13 (0.35-3.69)	.84	0.98 (0.30-3.27)	.98

Abbreviations: CI, confidence interval; HR, hazard ratio

[a] HRs were calculated using Cox proportional hazards models with age as the time scale. The models were also adjusted for history of anemia of any type present at baseline (any ICD-9 diagnosis code 280.x-285.x before index date).

© 2021 Huo N et al. *JAMA Network Open.*

[b] HRs were calculated using Cox proportional hazards models with age as the time scale and adjusted using inverse probability weights derived from a logistic regression model The models were also adjusted for history of anemia of any type present at baseline (any ICD-9 diagnosis code 280.x-285.x before index date). Interactions by age, indication, and estrogen therapy were assessed using separate models. No significant interactions were found.

[c] Cumulative risk of restless legs syndrome at 20 years after bilateral oophorectomy (or index) calculated using the Kaplan-Meier method. The estimates were adjusted using inverse probability weights derived from a logistic regression model.

[d] The benign condition (eg, benign tumor, cysts, endometriosis) was listed by the gynecologist in the medical record at the time of bilateral oophorectomy, but may not have been the sole indication for the surgery.

[e] Women without a benign ovarian condition. Historically, the terms *prophylactic*, *elective*, or *incidental* bilateral oophorectomy were used; however, we did not use these terms.

[f] Women who were receiving systemic estrogen therapy (only oral or transdermal) on their 46th birthday, after bilateral oophorectomy. Women who died or were lost to follow-up prior to their 46th birthday, or had not reached age 46 years as of December 31, 2014 were not included in this analysis. Follow-up for these analyses was started when women reached age 46 years.

[g] Women who were receiving systemic estrogen therapy (only oral or transdermal) on their 50th birthday, after bilateral oophorectomy. Women who died or were lost to follow-up prior to their 50th birthday, or had not reached age 50 years as of December 31, 2014 were not included in this analysis. Follow-up for these analyses was started when women reached age 50 years.

© 2021 Huo N et al. *JAMA Network Open.*

eFigure. Balance of Characteristics at Baseline Obtained Using Inverse Probability Weights

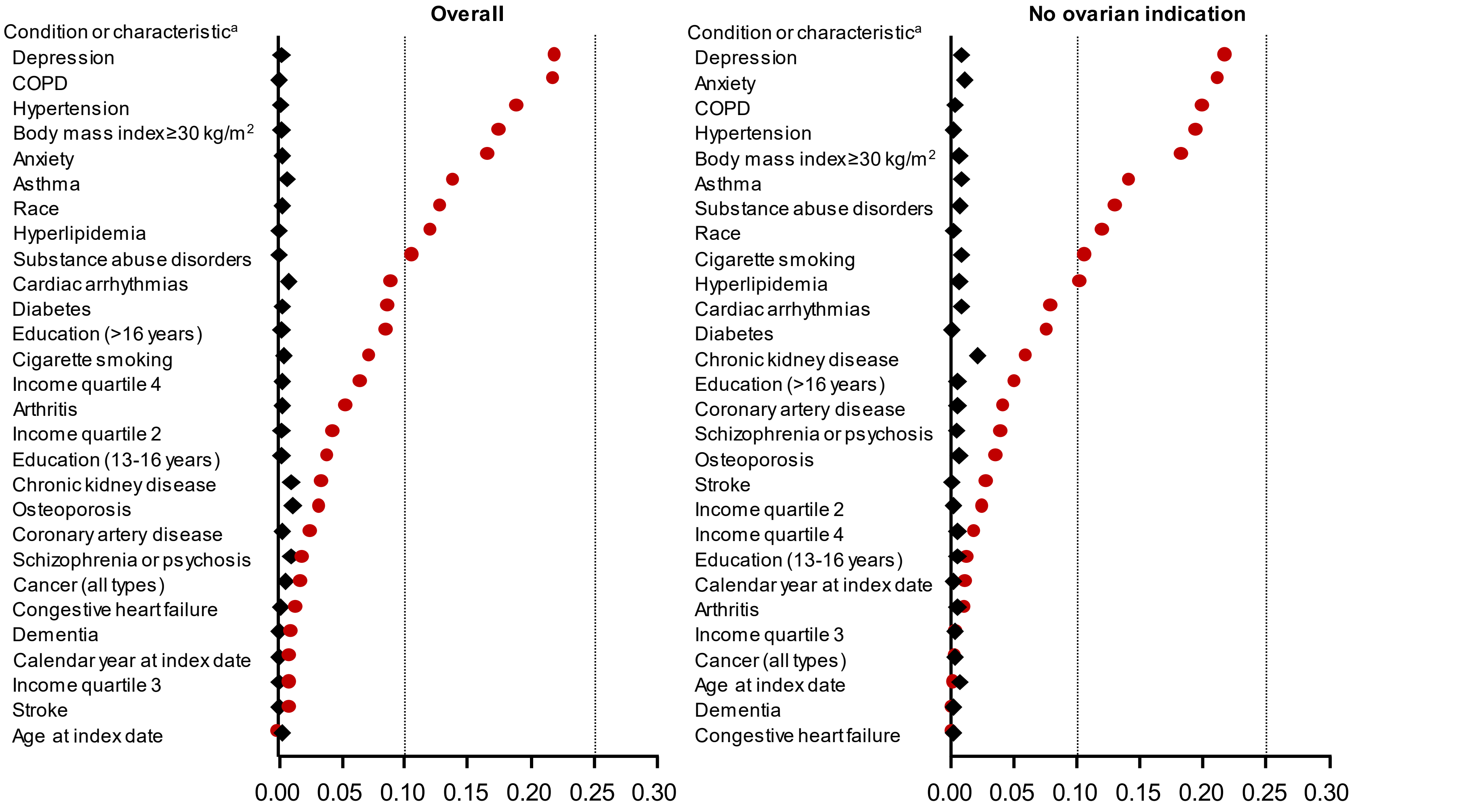

Abbreviations: COPD, chronic obstructive pulmonary disease; IPW, inverse probability weights.
The red circles indicate the absolute standardized differences before balancing and the black diamonds indicate these differences after balancing using IPW. After the IPW adjustment, all of the standardized differences were below the recommended threshold of 0.10, denoting negligible imbalance of these characteristics between the women with and without bilateral oophorectomy. For each condition or characteristic, the absolute standardized difference is defined as the absolute value of the difference in means for that characteristic between women in the 2 groups, divided by the pooled standard deviation for that characteristic. The weights were derived from propensity scores estimated from logistic regression models including 18 chronic conditions present at baseline, years of education (≤12, 13 to 16, >16), quartiles of household income (<$42 000, $42 000-56 999, $57 000-71 999, ≥$72 000), race (white vs nonwhite), body mass index (<30 vs ≥30 kg/m^2), cigarette smoking (current or former vs never), age at index date (continuous), and calendar year at index date (continuous). These models were fit overall, and separately in each stratum to maximize the balance at the index date. Weights greater than 10 were trimmed by setting these weights to the value of the 99th percentile for their respective group (with or without bilateral oophorectomy). The weights were then stabilized to reduce variability by dividing each weight by the mean weight for their respective group. After stabilization, the IPW ranged between 0.6 and 3.2

© 2021 Huo N et al. *JAMA Network Open.*

for the overall bilateral oophorectomy group and between 0.6 and 3.4 for the overall reference group. Among women who underwent bilateral oophorectomy for no ovarian indication and their age-matched referent women, the stabilized IPW ranged between 0.5 and 2.7 for the oophorectomy group and between 0.6 and 3.5 for the reference group.

© 2021 Huo N et al. *JAMA Network Open.*
